# Targeting the Heme-Heme Oxygenase System to Prevent Severe Complications Following COVID-19 Infections

**DOI:** 10.3390/antiox9060540

**Published:** 2020-06-19

**Authors:** Frank A. D. T. G. Wagener, Peter Pickkers, Stephen J. Peterson, Stephan Immenschuh, Nader G. Abraham

**Affiliations:** 1 Department of Dentistry-Orthodontics and Craniofacial Biology, Radboud Institute for Molecular Life Sciences, Radboud University Medical Center, Philips van Leydenlaan 25, 6525EX Nijmegen, The Netherlands; 2 Department of Intensive Care Medicine, Radboud Institute for Molecular Life Sciences, Radboud University Medical Center, 6500HB Nijmegen, The Netherlands; peter.pickkers@radboudumc.nl; 3 Department of Medicine, Weill Cornell Medicine, New York, NY 10065, USA; stp9039@nyp.org; 4 Institute for Transfusion Medicine, Hannover Medical School, 30625 Hannover, Germany; Immenschuh.Stephan@mh-hannover.de; 5 Departments of Medicine and Pharmacology, New York Medical College, Valhalla, NY 10595, USA; NADER_ABRAHAM@NYMC.EDU

**Keywords:** heme, heme oxygenase, SARS-CoV-2, inflammation, COVID-19

## Abstract

SARS-CoV-2 is causing a pandemic resulting in high morbidity and mortality. COVID-19 patients suffering from acute respiratory distress syndrome (ARDS) are often critically ill and show lung injury and hemolysis. Heme is a prosthetic moiety crucial for the function of a wide variety of heme-proteins, including hemoglobin and cytochromes. However, injury-derived free heme promotes adhesion molecule expression, leukocyte recruitment, vascular permeabilization, platelet activation, complement activation, thrombosis, and fibrosis. Heme can be degraded by the anti-inflammatory enzyme heme oxygenase (HO) generating biliverdin/bilirubin, iron/ferritin, and carbon monoxide. We therefore postulate that free heme contributes to many of the inflammatory phenomena witnessed in critically ill COVID-19 patients, whilst induction of HO-1 or harnessing heme may provide protection. HO-activity not only degrades injurious heme, but its effector molecules possess also potent salutary anti-oxidative and anti-inflammatory properties. Until a vaccine against SARS-CoV-2 becomes available, we need to explore novel strategies to attenuate the pro-inflammatory, pro-thrombotic, and pro-fibrotic consequences of SARS-CoV-2 leading to morbidity and mortality. The heme-HO system represents an interesting target for novel “proof of concept” studies in the context of COVID-19.

## 1. COVID-19 Infection May Result in Severe Inflammatory Complications

SARS-CoV-2 is causing a pandemic health issue, affecting millions of people and resulting in high morbidity and mortality. This virus is thought to specifically enter cells expressing the angiotensin-converting enzyme-2 (ACE2) receptors at their cell surface, such as cells in the nose, lungs, intestines, and kidneys [1,2,3]. Major common symptoms include fever, a dry cough, dyspnea, fatigue, and myalgia [4]. Minor common symptoms include expectoration, anorexia, chest tightness, nausea and vomiting, headache, pharyngalgia, shivering, and rhinorrhea [4].

Shortly following invasion of SARS-CoV-2 into the lungs, excessive pulmonary edema occurs as a consequence of vascular leakage. Disruption of the alveolar–epithelial barrier hampers optimal gas exchange [5], resulting in dyspnea. The majority of hospitalized Covid-19 patients also experience acute respiratory distress syndrome (ARDS), which appears to have various clinical features different from typical ARDS [6,7]. This is accompanied by other problems, including vascular inflammation, leukocyte recruitment, tissue injury, and the increased expression of interleukin-6, C-reactive protein, ferritin, and tissue factor [8]. In addition to these inflammatory insults, fibrin formation, microthrombi, and angiopathy develop that may result in vascular obstruction and fibrosis [9,10]. In addition to pulmonary damage, other organs with ACE2-positive cells may be affected, causing gastro-intestinal problems, multi-organ damage, and can ultimately even result in death.

In the absence of a vaccine against SARS-CoV-2, treatment is mainly supportive. In the meantime, we should develop hypotheses and perform “proof of principle” studies aimed at preventing or attenuating the complications leading to severe morbidity and death.

## 2. Can Injury-Derived Free Heme Contribute to COVID-19 Pathogenesis by Promoting Inflammation, Vascular Permeabilization and Thrombosis?

Heme is the functional group of a variety of heme–proteins, including cytochromes and hemoglobin (Hb), and is therefore crucial for many different cellular processes [11]. Excess of free heme has been shown to exacerbate and contribute to the pathogenesis of a wide variety of inflammatory diseases and conditions, such as sepsis, malaria, sickle cell disease, kidney disease, and multi-organ failure [11,12,13,14,15]. Additionally, within the lungs, free Hb and heme may be detrimental [16]. Chronic obstructive pulmonary disease (COPD) patients show increased cell-free Hb, correlating with disease severity [17]. In addition, ARDS patients exhibit alveolar hemorrhage and high levels of erythrocytes, hemolysis, and Hb in their pulmonary edema fluid [16,18,19,20,21]. Unfortunately, it is still not well understood how these erythrocytes enter the alveolar space [16]. This could occur via active transport, by increased endothelial and epithelial permeability, or by pronounced local vascular injury [16].

COVID-19 patients suffering from ARDS are critically ill and show also signs of hemolysis [22,23]. SARS-CoV-2 causes lung injury, resulting in death of inflammatory cells and sloughing of epithelial alveolar cells, the pulmonary vasculature, and hemolysis. Other COVID-19 patients display additional signs of tissue injury such as hemoptysis [24] or rhabdomyolysis [25], which may cause further cellular damage and release of heme-proteins and accumulation of free heme.

This made us to postulate that hemolysis-derived heme could initiate or contribute to many of the inflammatory phenomena witnessed in critically ill COVID-19 patients.

We and others previously demonstrated that excess free heme promotes oxidative and inflammatory stress [26,27,28,29], activates the vascular endothelium, and increases adhesion molecules and leukocyte recruitment [28,30]. Using radiolabeled liposomes, we further demonstrated that free heme causes vascular permeabilization resulting in edema [28]. Accumulated free heme and Hb may thus act as pathophysiologic mechanisms mediating pulmonary permeability and inflammation [31,32]. Alveolar fluid clearance is hampered in ARDS and also likely in hospitalized COVID-19 patients. Intratracheal administration of heme causes alveolar-capillary barrier dysfunction, and increases alveolar permeability, contributing to acute lung injury in mice [19]. Heme was shown to increase pulmonary edema by inhibiting amiloride-sensitive epithelial Na+ channel (ENaC)-activity, which plays a crucial role in sodium transport and fluid reabsorption in the lung [33].

Coagulation abnormalities, complement activation, endotheliitis, and thrombosis occur frequently in COVID-19 patients [34,35,36]. Interestingly, heme also promotes platelet activation, complement activation, vasculitis, and thrombosis [28,37,38,39]. Heme was recognized to act as a danger signal, damage-associated molecular pattern (DAMP), or alarmin [11,30,40,41,42] and was shown to activate Toll-like receptor 4 (TLR4) signaling [43]. Free heme promotes also oxidative stress by catalyzing the Fenton reaction [44,45], scavenges nitric oxide (NO) [46], and activates the inflammasome via TLR4 and NLR family pyrin domain containing 3 (NLPR3) [47,48]. In addition to heme promoting the expression of inflammatory cytokines [49], it potentiates tumor necrosis factor (TNF)-alpha induced inflammatory events and apoptosis [50].

The deleterious actions of heme could explain many of the observed manifestations during SARS-CoV-2 infection, including the increased capillary leakage resulting in pulmonary edema, vasculitis, leukocyte recruitment, and thrombus formation.

## 3. Protective Mechanisms against Free Heme

In order to attenuate these heme-induced pro-inflammatory, pro-oxidative, and pro-thrombotic actions, our body is normally equipped with different defense mechanisms. Following hemolysis, Hb is released that can be scavenged by serum haptoglobin (Hp). Free Hb outside the erythrocyte will turn into methemoglobin, which readily liberates its heme group [51]. Normally, this free heme gets scavenged by hemopexin (hpx) to prevent its injurious actions [52,53]. In case of severe hemolysis, or in blood clots, the hemoglobin and heme scavengers may be overwhelmed, exhausted, or physically not able to interact and neutralize free Hb and heme.

Alternative protective mechanisms against free heme are then pivotal for cellular survival. When heme enters the cell, it can modify proteins, DNA, and lipids [16,54,55]. Heme can also be transported out of the cell by Breast Cancer Resistance Protein (BCRP) that increases its chance of survival [56]. Finally, heme can be intracellularly degraded by heme oxygenase (HO) into biliverdin, iron, and carbon monoxide (CO). Biliverdin is then directly converted into the antioxidant bilirubin by biliverdin reductase (BVR), whilst iron gets scavenged by co-induced ferritin [11]. Heme oxygenase activity causes resolution of inflammation. We and others previously demonstrated that HO-activity is pivotal for reducing heme-induced vascular adhesion molecule expression and leukocyte extravasation, whereas inhibition of HO-activity further increases adhesion molecules and leukocyte influx [14,28,30,57,58,59].

The HO-effector molecules biliverdin/bilirubin, CO, and ferritin have each shown to be beneficial. Bilirubin signaling mediates protection against a variety of inflammatory diseases [60]. Increasing bilirubin has also been shown to increase the antioxidant capacity of the serum [61,62] and may protect against the oxidative properties of heme in a similar fashion as ascorbic acid possibly improves the condition of some COVID-19 patients [22].

Carbon monoxide signaling has been shown to protect the lung from inflammatory and oxidative insults and modulates autophagy, mitochondrial biogenesis, apoptosis, and cellular proliferation. [63]. CO downregulates both innate and cell mediated immunity, and approaches with CO-releasing molecules have been shown highly effective in animal models of T-cell mediated autoimmune diseases [64,65]. Genetic polymorphisms determining the protective response against hemoglobin/heme may contribute to differential disease outcomes. For example, a polymorphism in the HO-1 promoter determines the level of HO-1 expression following stress [66,67,68]. Individuals with longer (GT)n repeats have a lower transcriptional activity when compared to individuals with shorter (GT)n repeats, and have lower HO-1 levels and less protection against inflammatory insults [67]. It would be interesting to determine whether this differential protection by the antioxidant enzyme HO-1 has impact on the clinical outcome of SARS-CoV-2 infection. In addition, haptoglobin polymorphisms may result in more or less potent hemoglobin scavengers [69]. A decreased antioxidant capacity associated with the Hp 2-2 isoform, results in an increased risk of heme-induced inflammatory complications [70].

## 4. How Can We Protect against the Injurious Actions of Free Heme During SARS-CoV-2 Infection?

**Figure 1:**
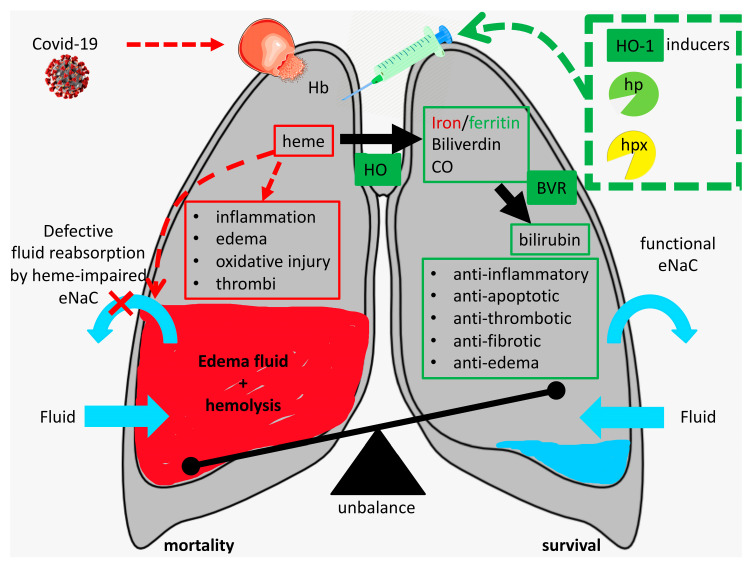
Heme-induced pulmonary complications following SARS-CoV-2 infection. Strategies to harness hemolysis-derived alveolar heme include administration of inducers of heme oxygenase (HO)-1 and hemoglobin (Hb)/heme scavengers (see green dashed box). “Proof of principle” studies should be performed to assess whether targeting heme by induction of HO-1 or administration of scavengers haptoglobin (hp)/hemopexin (hpx) can indeed attenuate or prevent complications in critically ill COVID-19 patients (see text for details).

Critically ill COVID-19 patients will often have excess of heme–proteins and heme in their alveoli thereby fueling exudate formation, platelet activation, inflammation, and fibrosis (see Figure 1). How can we prevent or attenuate these pulmonary complications?

In a mouse model of ARDS, heme-induced pulmonary edema, endoplasmic reticulum stress, and fibrosis could be attenuated by intramuscular administration of the heme-scavenger hemopexin while lung function improved [17,33,71]. In addition, induction of HO-1 was shown to decrease heme-induced edema and inflammation in this and other models [11,28,30,57,71,72]. Hb and heme scavengers (hp and hpx, respectively), HO-1 induction, and HO-effector molecules have already demonstrated to mediate potent protection against heme-induced inflammation, thrombosis, and fibrosis in diverse diseases [13,14,53,73,74,75,76,77,78,79,80,81], whereas inhibition of HO-activity aggravates disease [28,82,83,84]. Administration of Hb/heme scavengers, induction of HO-1, or HO-effector molecules could thus be beneficial to prevent or treat the injurious actions of heme (see Figure 1). The cytoprotective enzyme HO-1 can be induced by a wide spectrum of agents, including aspirin, statins, probucol, valsartan, niacin, resveratrol, and curcumin that could be safely used in humans [11,73,83,85].

Alternatively, Nrf2 is a transcriptional factor that induces several antioxidant protective target genes, among which is HO-1. Dimethyl fumarate (DMF) is a clinically used Nrf2 activator [86] that could possibly be used to prevent the many heme-induced complications during SARS-CoV-2 infection, such as edema, inflammation, and thrombosis and fibrosis by induction of the versatile HO-1 enzyme. Intriguingly, HO-1 induction and its effector molecules, CO and biliverdin/bilirubin, not only protect against inflammation, but have also potent antiviral properties that may be beneficial for fighting COVID-19 [87,88,89,90,91,92,93,94,95]. Preclinical in vitro and in vivo studies to eventual antiviral effects of HO1 inducers, bilirubin, or CO on Sars-CoV2 are thus warranted to better understand the possible translation of these concepts to the clinical setting.

Obesity, diabetes, chronic kidney disease, cardiovascular diseases, COPD, male sex, and aging form risk factors for developing severe complications when infected with SARS-CoV-2 [96,97,98,99]. These predisposing conditions, and inflammation in general, downregulate HO-1 expression and activity [67,74,100,101,102,103,104,105,106], further supporting that this compromised protection and diminished tolerance against inflammatory and oxidative stress promotes adverse clinical outcome in COVID-19 patients.

Recently, controversial and conflicting reports on possible associations between smoking and COVID-19 survival were reported [107]. Initially, increased risk was reported [107], which could be easily explained by the many well-established adverse health effects of smoking in, for instance, the lungs and vasculature. Surprisingly, recent (prepublished reports) studies, however, suggest that smokers may be under-represented among the COVID-19 patients with more severe disease. The smoking prevalence was lower than expected among hospitalized patients in diverse countries, suggesting that, counterintuitively, smoking could protect from severe complications [97,108,109,110]. This resulted in immediate investigations to the putative protective effects of nicotine against COVID-19 [111]. Although interesting, another intriguing possibility is glooming: not nicotine but the increased vascular carbon monoxide (CO) levels within smokers could possibly be the protective component. CO interacts with hemoglobin to form carboxyhemoglobin, which is thereby protected from the release of debilitating heme as previously also demonstrated for malaria [12].

Recently, the glucocorticoid dexamethasone was found to save many severely ill COVID-19 patients [112]. Since dexamethasone reduces hemolysis and induces HO-1 in macrophages [113], it is tempting to speculate that this increased protection against free heme attenuates the severity of disease in COVID-19 patients.

## 5. Conclusions

Until a vaccine against SARS-CoV-2 becomes available, we need to explore novel strategies to attenuate the pro-inflammatory, pro-thrombotic, and pro-fibrotic consequences of SARS-CoV-2 leading to morbidity and mortality. With this manuscript, we aimed to stress that heme is a likely culprit that may play a relevant role in initiating and contributing to the pulmonary complications displayed in critically ill COVID-19 patients. Inducing HO-1 expression or administration of Hb/heme scavengers or HO-effector molecules may prevent SARS-CoV-2-induced pulmonary complications by its antiviral, anti-inflammatory, antithrombotic, and antifibrotic activities. The heme-HO system represents an interesting target for novel “proof of concept” studies in the context of COVID-19.
